# Vitamin K: Metabolism, Genetic Influences, and Chronic Disease Outcomes

**DOI:** 10.1002/fsn3.70431

**Published:** 2025-06-17

**Authors:** Montana Dupuy, Nicola P. Bondonno, Pratik Pokharel, Allan Linneberg, Itamar Levinger, Carl Schultz, Jonathan M. Hodgson, Marc Sim

**Affiliations:** ^1^ Nutrition & Health Innovation Research Institute, School of Medical and Health Sciences Edith Cowan University Perth Western Australia Australia; ^2^ The Danish Cancer Institute Copenhagen Denmark; ^3^ Center for Clinical Research and Prevention Copenhagen University Hospital, Bispebjerg and Frederiksberg Frederiksberg Denmark; ^4^ Department of Clinical Medicine, The Faculty of Health and Medical Sciences University of Copenhagen Copenhagen Denmark; ^5^ Institute for Health and Sport (IHES) Victoria University Melbourne Victoria Australia; ^6^ Department of Cardiology Royal Perth Hospital Perth Western Australia Australia; ^7^ Medical School The University of Western Australia Perth Western Australia Australia; ^8^ Royal Perth Hospital Research Foundation Perth Western Australia Australia

**Keywords:** bone health, cancer, cardiovascular disease, diabetes, vitamin K1, vitamin K2

## Abstract

Vitamin K refers to a group of lipid‐soluble vitamins that exist in two natural isoforms; phylloquinone (PK, vitamin K1) and menaquinones (MKs, vitamin K2). Phylloquinone, the primary dietary source, is found abundantly in green vegetables and plant oils. Menaquinones (MK‐4 through MK‐13) are synthesized by anaerobic bacteria and may be obtained through the diet from fermented foods and animal products (e.g., meats, dairy and eggs). Originally recognized for its role in blood coagulation, vitamin K is an essential cofactor for the posttranslational carboxylation of vitamin K‐dependent proteins (VKDPs), which are implicated in various physiological processes including; blood coagulation, calcium homeostasis, as well as metabolic and inflammatory pathways. Therefore, vitamin K has attracted considerable research interest for its potential implications in several diseases. While promising, the specific roles of vitamin K in various health conditions, the quantity of vitamin K (both PK and MKs) required for the function of various VKDPs, and the influence of genetics on vitamin K metabolism, remain unclear. This review aims to (i) provide an overview of the structure, dietary sources, metabolism, and physiological roles of vitamin K, including those relating to; cardiovascular diseases, type 2 diabetes, respiratory conditions, musculoskeletal health and cancer; (ii) discuss the impact of genetic factors on vitamin K status and how such factors modulate the role of vitamin K in the aforementioned chronic diseases; and (iii) outline key directions for future research.

## Graphical abstract

Vitamin K exists in two natural isoforms: phylloquinone (PK, vitamin K1) and menaquinones (MKs, vitamin K2), and is an essential cofactor for the post‐translational carboxylation of vitamin K‐dependent proteins (VKDPs). VKDPs are implicated in various physiological processes, including blood coagulation, calcium homeostasis, as well as metabolic and inflammatory pathways. Therefore, vitamin K has attracted considerable research interest for its potential implications in several diseases (e.g., cardiovascular diseases, type 2 diabetes, cancer, musculoskeletal health and respiratory conditions). While promising, the specific roles of vitamin K in these health conditions, the quantity of vitamin K (both PK and MKs) required for the function of various VKDPs, and the influence of genetics on vitamin K metabolism remain unclear. Created in BioRender. BioRender.com/v78q815.

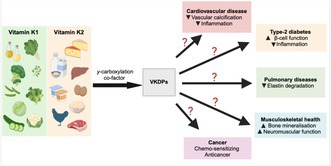

## 1 Introduction

Vitamin K is a naturally occurring, lipid‐soluble vitamin that was originally recognized for its essential role in blood coagulation (Mladěnka et al. 2022). The term “vitamin K” refers to a group of compounds that function primarily as essential cofactors for the carboxylation of vitamin K‐dependent proteins (VKDPs) (Barrett et al. 2018; Mladěnka et al. 2022). Two sub‐classifications of most VKDPs have been identified; hepatic VKDPs are involved in coagulation, whereas the physiological roles VKDPs found in extrahepatic tissues, including; bone, cartilage and vascular smooth muscle cells, extend far beyond coagulation (Mladěnka et al. 2022; Wen et al. 2018). Through activation of these extrahepatic VKDPs, vitamin K has been shown to play a role in bone homeostasis and vascular calcification processes, as well as in metabolic and inflammatory pathways (Halder et al. 2019; Mladěnka et al. 2022). These findings are promising and indicate potential implications for vitamin K across numerous health outcomes. However, the specific roles of vitamin K in various clinical and subclinical conditions remains underexplored and/or ambiguous (Halder et al. 2019; Mladěnka et al. 2022).

Consolidation of the current evidence is warranted to identify fundamental knowledge gaps and direct future research. As such, this review; (i) provides an overview of the structure, dietary sources, metabolism, and physiological roles of vitamin K, including those relating to; cardiovascular diseases, type 2 diabetes, respiratory conditions, musculoskeletal health and cancer; (ii) discusses the impact of genetic factors on vitamin K status and how such factors modulate the role of vitamin K in the aforementioned chronic diseases, and; (iii) outlines key directions for future research.

### 1.1 Vitamin K Structure

**FIGURE 1:**
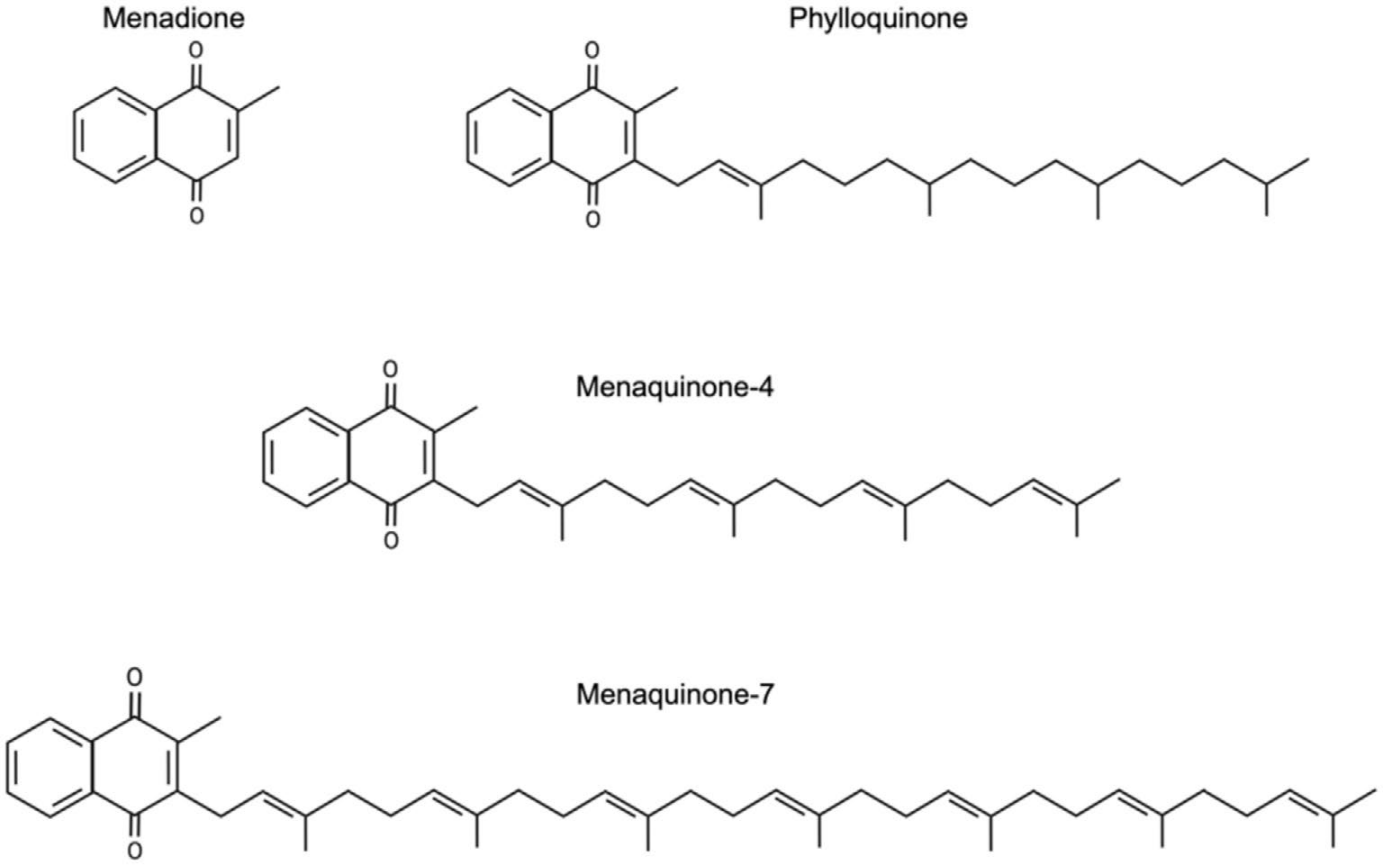
Chain structures of vitamin K1 (phylloquinone [PK]), two vitamin K2 isoforms (menaquinone 4 [MK‐4] and menaquinone 7 [MK‐7]), and vitamin K3 (menadione). Created in BioRender. Dupuy, M. (2024) BioRender.com/m97w819.

Vitamin K exists in two natural isoforms, phylloquinone (PK, vitamin K1) and menaquinone (MK, vitamin K2), which share a common 2‐methyl‐1,4‐naphtoquinone ring structure, called menadione (Mladěnka et al. 2022; Palmer et al. 2020). Phylloquinone is synthesized by photosynthetic plants and algae, and contains a phytyl side chain of four isoprenoid units, one of which is unsaturated (Figure 1) (Shearer and Newman 2008). Menaquinones are a group of isoprenologs, each with a side chain of unsaturated isoprenoid units that vary in length (Figure 1) (Halder et al. 2019; Shearer and Newman 2008). The name of each MK is derived from the number of repeated units on the side chain, ranging from four through 13 (MK‐4 to MK‐13) (Halder et al. 2019; Shearer and Newman 2008). While each MK is similar in structure, their origin and synthesis varies (Mladěnka et al. 2022; Walther et al. 2013). Specifically, MK‐4 can be synthesized from PK in humans and animals, while MK‐5 through MK‐13 are synthesized anaerobically by bacteria (Mladěnka et al. 2022; Walther et al. 2013).

### 1.2 Sources of Vitamin K

**FIGURE 2:**
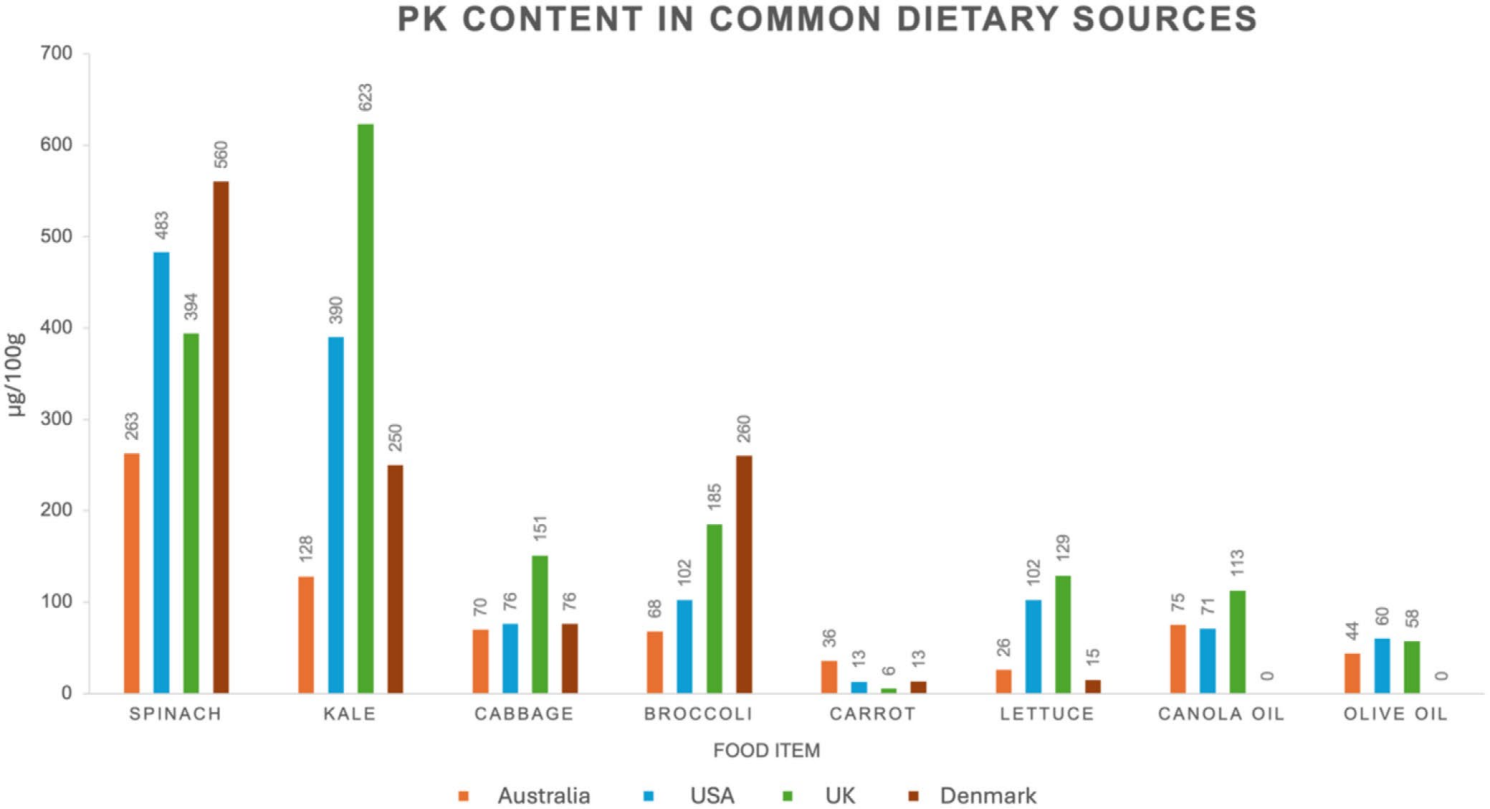
Phylloquinone content in common dietary sources based on data from four countries: Australia (Palmer, Koch, et al. 2021), the USA (U.S. Department of Agriculture n.d.), the United Kingdom (McCance and Widdowson 2014), and Denmark (National Food Institute, Technical University of Denmark 2024).

Phylloquinone is obtained by humans via dietary sources and is found abundantly in leafy green and cruciferous vegetables (such as spinach, kale, cabbage, and broccoli), plant oils (such as canola, soybean, and olive oils) and various herbs (such as parsley, mint, and marjoram) (Figure 2) (Palmer, Bellinge, et al. 2021; Damon et al. 2005; Presse et al. 2015; National Food Institute, Technical University of Denmark 2024). Phylloquinone is estimated to contribute ~75%–90% of total vitamin K intake (Mladěnka et al. 2022). The PK content in plant sources is known to vary regionally and seasonally (Damon et al. 2005). For example, the PK content in kale from the United Kingdom is reported to be up to almost five times higher (~159%–486%) than that reported within kale from other origins (Figure 2) (McCance and Widdowson 2014; Palmer, Koch, et al. 2021; U.S. Department of Agriculture n.d.; National Food Institute, Technical University of Denmark 2024). This variance is suggested to be due to a multitude of factors, including climate conditions, soil quality, and plant maturation (Damon et al. 2005). Cooking and storage methods may also affect the PK content (Damon et al. 2005; Ferland and Sadowski 1992; Lee et al. 2018). For example, vitamin K (both PK and MKs) is relatively heat stable; thus, PK content is retained during the microwaving and steaming of vegetables (Damon et al. 2005; Lee et al. 2018) and may be further enriched using PK‐rich oils during cooking. However, PK is extremely sensitive to fluorescent lighting and sunlight, therefore the PK content in plant oils stored in exposed conditions is decreased (Ferland and Sadowski 1992).

**FIGURE 3:**
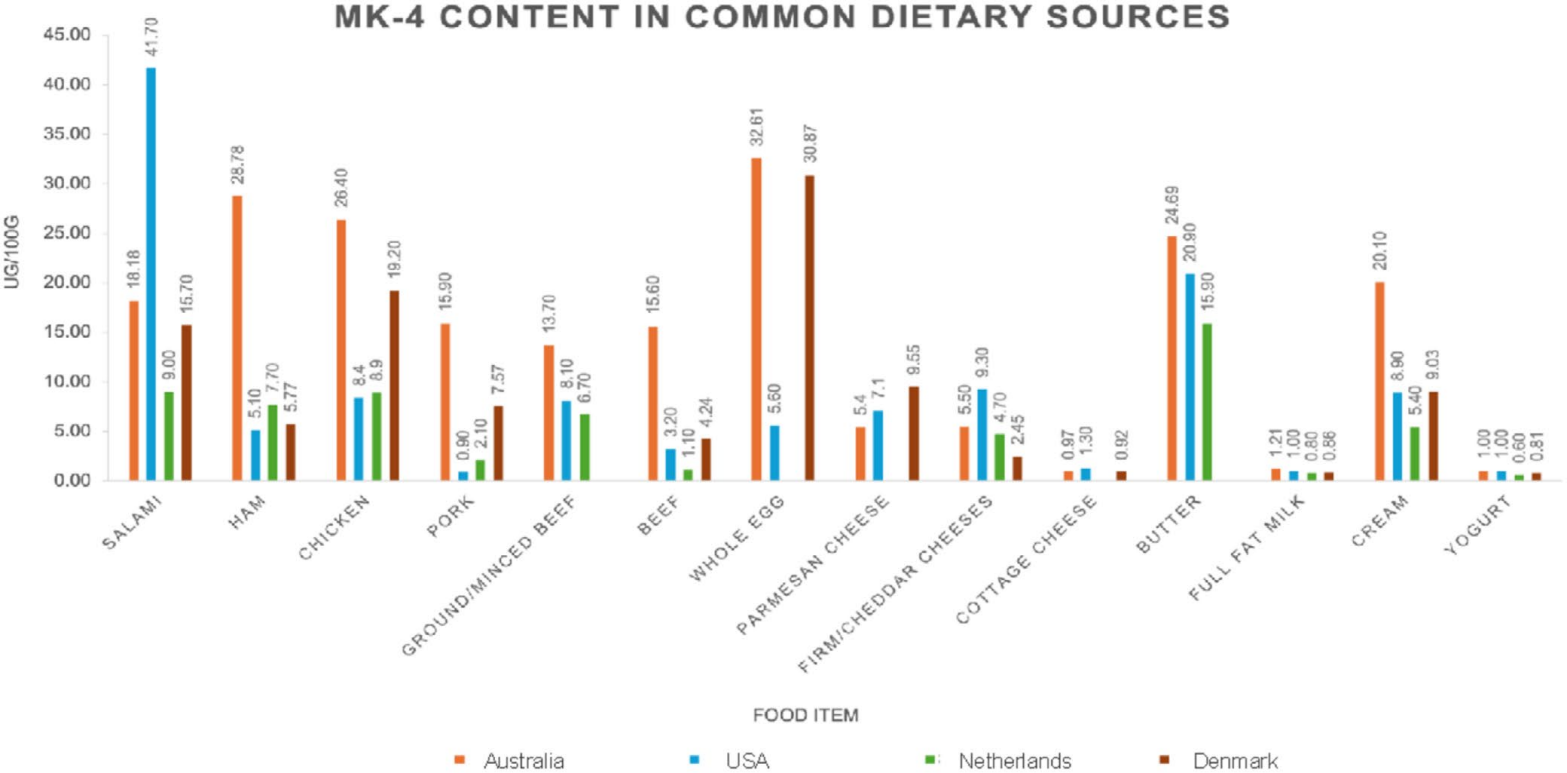
Menaquinone‐4 content in common dietary sources based on data from five published databases, from four different regions: Australia (Palmer, Koch, et al. 2021), the USA (U.S. Department of Agriculture n.d.), the Netherlands (Schurgers and Vermeer 2000), and Denmark (National Food Institute, Technical University of Denmark 2024; Jensen et al. 2025). Values were left blank where no data was available.

Menaquinones are also obtained via dietary sources from fermented foods, dairy, eggs and animal meats, and may be endogenously synthesized by bacteria in the intestine (Mladěnka et al. 2022). However, the concentration of each form of MK varies among dietary sources (Mladěnka et al. 2022; Schurgers and Vermeer 2000). As MK‐4 is synthesized from PK in animals, many animal products are rich dietary sources (e.g., chicken, beef, eggs, and cream) (Figure 3) (Mladěnka et al. 2022; Palmer, Bellinge, et al. 2021; Schurgers and Vermeer 2000). While MK‐4 content within these foods is characteristically high, the specific content reported in published data sources is varied. For example, the MK‐4 content reported in Australian beef is higher (~367%–1418%) than the MK‐4 content reported in beef from the Netherlands, the USA and Denmark (Figure 3) (Palmer, Koch, et al. 2021; Schurgers and Vermeer 2000; U.S. Department of Agriculture n.d.; National Food Institute, Technical University of Denmark 2024; Jensen et al. 2025). Yet, the MK‐4 content within full fat milk and yogurt products from these countries is reportedly similar (Figure 3). While the MK‐4 content in foods sourced from four differing regions are presented in Figure 3, minimal data are available for the MK‐4 content of many commonly consumed foods, across a broad range of regions. The longer chain MKs (MK‐5 through MK‐13) are found abundantly in fermented foods, due to their synthesis by anaerobic bacteria (Mladěnka et al. 2022; Schurgers and Vermeer 2000). For example, cheese is the primary food source for many long chain MKs (e.g., MK‐7, MK‐8, MK‐9 and MK‐11) (Fu et al. 2017; Jensen et al. 2021; Schurgers and Vermeer 2000), while MK‐7 is also found abundantly in Nattō (fermented soybeans) (Palmer, Koch, et al. 2021; Schurgers and Vermeer 2000). It must be noted that comprehensive data on the MK content of foods remains scarce globally, especially for the longer chain MKs (Lyytinen and Linneberg 2023; Mladěnka et al. 2022). Furthermore, little is known on the regional variance of the MK content of food, nor on the effects of cooking and storage methods (Lyytinen and Linneberg 2023; Mladěnka et al. 2022). Thus, estimating dietary MK intakes remains complicated and is a key barrier to researching potential health benefits associated with this vitamin K isoform.

Supplemental and synthetic forms of vitamin K also exist. Both PK and MK can be chemically synthesized and used pharmaceutically or as a dietary supplement (Simes et al. 2020). Supplemental forms of PK are not known to be harmful or toxic in humans (Mladěnka et al. 2022; Simes et al. 2020); therefore, they may be attractive, particularly within research and clinical settings. Vitamin K3 (menadione) is a synthetic form of vitamin K with no side chain (Figure 1) (Mladěnka et al. 2022; Shearer and Newman 2014). Contrary to supplemental forms of PK and MK, in high doses menadione is known to be toxic in a dose‐dependent manner, which may result in hemolytic anemia, especially in infants (Mladěnka et al. 2022; Shearer and Newman 2014). Vitamin K3 is not derived from dietary sources, yet acts as a metabolic intermediate (Hirota et al. 2013; Mladěnka et al. 2022; Shearer and Newman 2014; Thijssen et al. 2006). Vitamin K3 is rarely described in nutritional literature; therefore, this review primarily discusses the natural, dietary vitamin K forms (i.e., PK and the MKs). For more detail on vitamin K3, the reader is directed elsewhere (Hirota et al. 2013; Shearer and Newman 2014; Thijssen et al. 2006).

### 1.3 Bioavailability, Absorption and Metabolism

The bioavailability, absorption, and metabolism of vitamin K has been comprehensively reviewed previously (Mladěnka et al. 2022; Shearer et al. 2012; Shearer and Newman 2008, 2014). In brief, vitamin K bioavailability differs between the isoforms (Schurgers and Vermeer 2000). Specifically, as dietary PK is often tightly bound within plant chloroplasts, it is considerably less bioavailable than dietary MKs, yet is enhanced, by up to three times, when ingested in the co‐presence of lipids (Gijsbers et al. 1996; Shearer et al. 2012). Little is known about the absorption of endogenously synthesized MKs (Shearer et al. 2012). However, dietary MKs are highly bioavailable, particularly in the case of the long chain MKs (Schurgers and Vermeer 2000). This may be due, at least in part, to the high fat nature of the primary MK food sources, such as cheese (Fu et al. 2017). Dietary fat consumption results in bile secretion into the small intestine, a requirement for absorption of lipid‐soluble vitamins, including vitamin K. Vitamin K absorption follows an established pathway involving; incorporation of vitamin K and bile salts into micelles, enterocyte uptake, chylomicron binding, and entry into the bloodstream via lymphatic circulation, as described extensively elsewhere (Mladěnka et al. 2022; Shearer et al. 2012; Shearer and Newman 2014). Vitamin K is primarily stored within adipose tissue, however is found in the liver and some extrahepatic organs (Lyytinen and Linneberg 2023; Mladěnka et al. 2022). As such, serum vitamin K levels are characteristically low, particularly for PK, which has a 1–2 h half‐life (Mladěnka et al. 2022; Schurgers and Vermeer 2000). The elimination half‐life of MKs in serum is varied, for example, MK‐9 has a ~60‐h half‐life (Mladěnka et al. 2022), compared to a longer period of ~3 days for MK‐7 (Mladěnka et al. 2022; Schurgers and Vermeer 2000). Collectively, the higher bioavailability and longer half‐life of MKs, compared to PK, may explain why clinical trials exploring vitamin K commonly adopt MKs (e.g., MK‐7).

### 1.4 Vitamin K Status Biomarkers

Various biochemical measures to assess vitamin K status have been identified, yet contrary to many other nutrients, a gold‐standard biomarker for vitamin K has not been established (Shea and Booth 2016). Serum PK is widely used within nutrition research as it is rapidly affected by acute dietary intake, peaking 6–10 h postprandially (Novotny et al. 2010; Shea and Booth 2016; Schurgers and Vermeer 2000). Conversely, few studies adopt circulating MKs as a nutritional biomarker of vitamin K status, likely due to the poor detection of MKs in circulation, unless supplemented or consumed in high quantities (Shea and Booth 2016).

Vitamin K availability governs the rate of posttranslational carboxylation of VKDPs (McCann and Ames 2009; Shea and Booth 2016). When vitamin K status is low, the proportion of inactive forms of VKDPs increases (McCann and Ames 2009; Shea and Booth 2016), which may also serve as surrogate markers of vitamin K status. For example, osteocalcin (OC), a VKDP synthesized primarily by bone, is detectable in serum (Gundberg et al. 2012; Shea and Booth 2016). Undercarboxylated OC (ucOC) levels vary in response to vitamin K intake (Gundberg et al. 2012; Sokoll et al. 1997). Specifically, when vitamin K intakes are inadequate, posttranslational carboxylation of OC is reduced, resulting in higher serum ucOC (Shea and Booth 2016; Sokoll et al. 1997). To account for osteoblastic synthesis, serum ucOC is routinely expressed in relation to total OC (tOC) as a percentage (%ucOC) or as a ratio (ucOC:tOC ratio), dependent on the assay used, when adopted to indicate vitamin K status (Gundberg et al. 2012; Shea and Booth 2016). Specifically, a higher %ucOC (or ucOC:tOC) reflects a lower vitamin K status. Another VKDP, matrix Gla protein (MGP) in its various forms (including the undercarboxylated forms; undercarboxylated MGP [ucMGP] and dephosphorylated ucMGP [dp‐ucMGP]) is detectable in plasma (Shea and Booth 2016). Posttranslational carboxylation and phosphorylation of MGP are governed by vitamin K (Roumeliotis et al. 2019; Schurgers et al. 2008; Shea and Booth 2016). Plasma dp‐ucMGP has been found to decrease in response to vitamin K supplementation and has been inversely associated with dietary PK intakes up to ~100–150 μg/day, from which point a plateau occurred (Shea and Booth 2016). As such, circulating ucMGP and dp‐ucMGP have also been suggested as biomarkers of vitamin K status (Roumeliotis et al. 2019; Schurgers et al. 2008; Shea and Booth 2016). For comprehensive detail on vitamin K status biomarkers and an evaluation of their use in nutrition and clinical research, the reader is directed to Shea and Booth (2016).

### 1.5 Physiological Roles of Vitamin K

**FIGURE 4:**
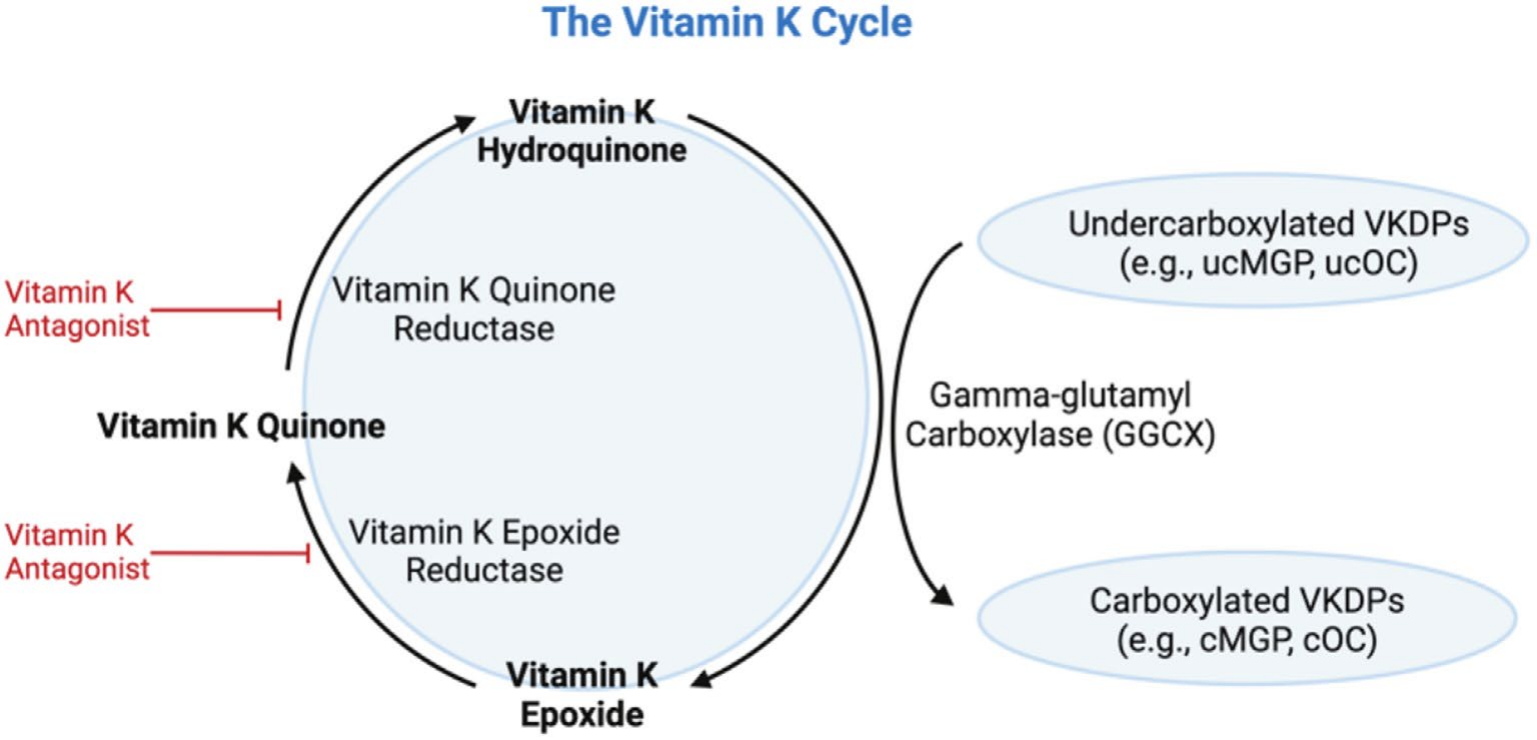
The vitamin K cycle. Created in BioRender. Dupuy, M. (2024) BioRender.com/p68j777.

Vitamin K (in its reduced form, hydroquinone) is an essential cofactor for the *y‐*carboxylation of VKDPs by the enzyme gamma‐glutamyl carboxylase (GGCX) (Lyytinen and Linneberg 2023; McCann and Ames 2009; Mladěnka et al. 2022; Stafford 2005). This results in posttranslational VKDP activation and formation of vitamin K epoxide (Lyytinen and Linneberg 2023; McCann and Ames 2009; Mladěnka et al. 2022; Stafford 2005). Vitamin K epoxide may be reduced to vitamin K quinone by the enzyme vitamin K epoxide reductase (VKOR) and recycled (Lyytinen and Linneberg 2023; McCann and Ames 2009; Mladěnka et al. 2022; Stafford 2005). In its entirety, this process is known as the vitamin K cycle (Figure 4), as described in greater depth elsewhere (Shearer and Newman 2014; Stafford 2005). Due to vitamin K recycling, the physiological requirements for vitamin K are considered to be low (McCann and Ames 2009; Mladěnka et al. 2022). The roles of hepatic VKDPs (including coagulation factors; II [prothrombin], VII, IX, and X, and proteins; C, S, and Z) in blood coagulation processes are well established (Mladěnka et al. 2022). Not as extensively understood are the diverse and complex functions of the various extrahepatic VKDPs (e.g., OC, MGP, Gla‐rich protein [GRP], and growth arrest specific protein 6 [Gas6]), which have gained more recent attention (Mladěnka et al. 2022; Wen et al. 2018). For example, MGP, ucOC, and GRP have been shown to be implicated in calcium homeostasis, glucose metabolism, and inflammatory pathways (Halder et al. 2019; Mladěnka et al. 2022; Smith et al. 2024; Willems et al. 2014).

The carboxylation rate of VKDPs is primarily governed by vitamin K availability (McCann and Ames 2009; Mladěnka et al. 2022), with low vitamin K status resulting in an elevated proportion of undercarboxylated VKDP forms (Lyytinen and Linneberg 2023; McCann and Ames 2009). Interestingly, the triage theory hypothesis suggests that in states of scarcity, available vitamin K is preferentially distributed for the *y‐*carboxylation of VKDPs with short‐term critical functions (e.g., coagulation factors II [prothrombin], VII, IX, and X) (McCann and Ames 2009). This results in lower available vitamin K for the *y‐*carboxylation of VKDPs implicated in broader, yet less critical, physiological processes (e.g., OC and MGP) (McCann and Ames 2009; Mladěnka et al. 2022). Given the diverse roles of VKDPs, optimizing vitamin K availability may be important for various health outcomes. However, the current evidence remains insufficient to derive specific dietary vitamin K requirement values for human health and the optimal functioning of VKDPs, beyond effects of coagulation (Shearer et al. 2012; Turck et al. 2017). For this reason, and notable complications in quantifying vitamin K intakes, the current global dietary recommendations for vitamin K are set as adequate intakes (AIs), which differ globally (Blomhoff et al. 2023; National Institutes of Health 2021; Turck et al. 2017). For example, in Europe and Nordic countries, 1 μg/kg/day of PK is recommended (Blomhoff et al. 2023; Turck et al. 2017), compared to 90–120 μg/day of total vitamin K in the United States (National Institutes of Health 2021). These are primarily set to prevent deficiency and/or provide sufficient vitamin K for hemostasis (Blomhoff et al. 2023; Lyytinen and Linneberg 2023; McCann and Ames 2009; National Institutes of Health 2021; Turck et al. 2017). The remaining ambiguity surrounding the quantity of vitamin K that is required for the function of various VKDPs, and therefore optimal human health outcomes, is one of the many limitations to setting more comprehensive dietary guidelines for vitamin K (Lyytinen and Linneberg 2023). Further, it remains unknown if the optimal amount of vitamin K differs between health outcomes (e.g., coagulation vs. bone or vascular health), an area which warrants further investigation.

### 1.6 Genetic Influences on Vitamin K Status

Genetic variants that influence vitamin K metabolism may provide insight into the large interindividual variability in vitamin K status that dietary intake alone cannot explain (Dashti et al. 2014; Hirai et al. 2015). Mendelian randomization (MR) studies, which use genetic data to explore causal relationship between risk factors and health outcomes (Burgess et al. 2013), are a valuable tool for investigating the role of vitamin K across health outcomes (Zwakenberg et al. 2019). Key genes of the vitamin K pathway, *VKORC1*, *CYP2C9*, and *CYP4F2* (Gong et al. 2011; Lee et al. 2002), also play pivotal role in determining warfarin doses (Takeuchi et al. 2009), a widely used vitamin K antagonist that works as an anticoagulant by targeting the VKOR enzyme (Booth and Suttie 1998; Yu et al. 2016). Mutations in *VKORC1* reduce the activity of the VKOR enzyme, impair vitamin K recycling, and necessitate lower warfarin doses. Similarly, *CYP2C9* variants (CYP2C9*2 and CYP2C9*3) decrease enzymatic activity, requiring reduced warfarin doses to achieve therapeutic anticoagulation effects. Together, *VKORC1* and *CYP2C9* polymorphisms account for approximately 40% of warfarin dose variability (Takeuchi et al. 2009). Variants in *CYP4F2*, an enzyme responsible for vitamin K oxidation, increase the hepatic vitamin K pool and consequently raise warfarin dose requirements (Zubiaur et al. 2024). Other candidate genes, such as *GGCX* (Sun et al. 2015) and *PROC* (Wadelius et al. 2007), have also been implicated in influencing warfarin dosing. The *GGCX* gene, critical for the carboxylation of VKDPs, has heterozygous group for single nucleotide polymorphisms (SNPs) that are significantly associated with lower %ucOC levels compared to homozygous group (Crosier et al. 2009). Consequently, variants in *GGCX* may impair the carboxylation activity, disrupt the vitamin K cycle and adversely affect vitamin K status (Crosier et al. 2009), leading to diminished clotting factor activation (Rost et al. 2004), and alterations in warfarin dose requirements.

The *APOE* gene plays a pivotal role in vitamin K metabolism by regulating lipoprotein‐mediated transport and hepatic uptake (Kohnke et al. 2005). The three major alleles exhibit differential effects on vitamin K uptake efficiency, with *E2* carriers demonstrating the lowest and *E4* carriers the highest clearance of vitamin K‐rich lipoproteins, which may lead to variations in serum vitamin K concentrations and hepatic availability (*E4* > *E3* > *E2*) (Kohlmeier et al. 1996; Saupe et al. 1993). These differences might influence warfarin dose requirements. While some studies suggest higher warfarin dose for *E4* carriers (Kimmel et al. 2008; Kohnke et al. 2005), others report lower dose needs (Visser et al. 2005). Interestingly, a recent systematic review indicated minimal effects of *APOE* genotypes on warfarin dosing, except for *E2* carriers, who may require reduced doses due to lower vitamin K uptake efficiency (Yu et al. 2016). These relationships appear to vary across ethnic groups, emphasizing the need for population‐specific investigations.

Genome Wide Association Studies (GWAS) have identified additional genetic determinants regulating vitamin K levels. Using genetic data from 2138 European individuals, researchers identified 11 SNPs on five independent loci within/near the candidate genes, including the *APOA1/C3/A4/A5* cluster (involved in lipoprotein metabolism), *COL22A1*, *CDO1*, *CTNAA2*, and *CYP4F2* (a PK oxidase) that were associated with circulating PK (Dashti et al. 2014). However, the precise role of certain genes, such as *COL22A1* and *CDO1*, in regulating vitamin K remains poorly understood. *COL22A1*, which encodes collagen, provides mechanical stability to the myotendinous junction (Ahmad et al. 2020), and may support extracellular matrix integrity in conjunction with VKDPs (Willems et al. 2014). *CDO1*, a key enzyme in cysteine catabolism, is involved in various physiological processes such as redox homeostasis and osteoblastic differentiation (Chen et al. 2023), and may impair vitamin K cycle enzymes under oxidative stress.

Additionally, SNPs in the MGP gene region have been linked to circulating inactive dp‐ucMGP levels, a biomarker of vitamin K status (Liu et al. 2015). Variants in this region influence vitamin K‐dependent processes, such as vascular calcification and bone mineralization (Cozzolino et al. 2010). However, it remains unclear whether these effects are independent of vitamin K, though these polymorphisms may not be uncommon. Collectively, these genetic variations highlight the importance of genotype‐informed approaches in understanding vitamin K metabolism, anticoagulant therapy, and the role of vitamin K in various health outcomes. Several studies employing the MR paradigm have examined associations between genetic predictors of vitamin K status, specifically circulating PK concentrations (Zwakenberg et al. 2020, 2019) and inactive MGP (Liu et al. 2015; Wei et al. 2018; Zwakenberg et al. 2020), and various health outcomes. These will be briefly discussed in relation to specific diseases that may benefit from vitamin K in subsequent sections below.

## 2 Vitamin K and Cardiovascular Disease

Atherosclerotic vascular diseases (ASVDs), including coronary heart disease (CHD) and stroke, remain global leading causes of death (World Health Organization 2023). Inflammation, endothelial dysfunction, and vascular calcification are common attributes of ASVDs (Cai and Harrison 2000; Chen and Moe 2012). The potential involvement of vitamin K in cardiovascular health may be attributed to the anti‐inflammatory properties and calcium binding affinity of various extrahepatic VKDPs. These include MGP, GRP, and Gas6 (Schurgers et al. 2008; Wen et al. 2018) that require activation via vitamin K‐dependent carboxylation processes (Roumeliotis et al. 2019; Schurgers et al. 2008; Wen et al. 2018). Vitamin K has been reported to suppress inflammation, likely by inhibiting the activation of the nuclear factor κΒ signaling pathway (Ohsaki et al. 2010). However, research in this area is limited to in vitro and animal studies (Ohsaki et al. 2010; Reddi et al. 1995). Observational human studies also support an inverse association between serum PK and inflammatory biomarkers, including IL‐6 and CRP (Shea et al. 2008, 2014), which are associated with increased risk of cardiovascular events (Libby et al. 2002). Considering the limited studies in humans to date, the potential effect of vitamin K on inflammation, and whether this relates to improved cardiovascular health, warrants further investigation.

Vascular calcification is characterized by hydroxyapatite mineral deposition within the intimal and medial layers of the arterial wall, resulting in atherosclerotic plaque formation and increased arterial stiffness (Chen and Moe 2012; Durham et al. 2018). Vascular calcification progression may be inhibited via the mineral binding affinity of carboxylated VKDPs (Schurgers et al. 2008; Wen et al. 2018), of which MGP has gained significant attention (Schurgers et al. 2008). Positive associations between inactive dp‐ucMGP and vascular calcification have been reported (Boxma et al. 2012; Dalmeijer, Van der Schouw, Vermeer, et al. 2013; Roumeliotis et al. 2019). As the proportion of inactive forms of ucMGP to active carboxylated MGP (cMGP) is governed by vitamin K, the ratios of circulating ucMGP or dp‐ucMGP to either cMGP or total MGP (tMGP) have been proposed as objective biomarkers of vitamin K status in humans (Dalmeijer, Van der Schouw, Vermeer, et al. 2013; Shea et al. 2011). A MR study reported a relationship between lower genetically predicted dp‐ucMGP concentrations, representing higher vitamin K status, and reduced CHD risk (Zwakenberg et al. 2020). A meta‐analysis also reported positive associations between dp‐ucMGP and risk of CVD events (pooled HR 1.57 95% CI: 1.19–2.06, from three studies [*n =* 1190] comparing the highest to lowest tertiles) (Chen et al. 2019). As higher vitamin K intakes are known to decrease dp‐ucMGP levels (Dalmeijer, Van der Schouw, Vermeer, et al. 2013; Shea et al. 2011), this may partly explain why green leafy vegetable consumption is typically associated with better cardiovascular health.

Randomized control trials (RCTs) exploring vitamin K supplementation have reported increased vascular elasticity (Braam et al. 2004), and slower progression of both coronary artery calcification (CAC) (Shea et al. 2009) and aortic valve calcifications (AVC) (Brandenburg et al. 2017). Conversely, a systematic review from 2020 (nine placebo‐controlled RCTs, all ≥ 12 weeks of intervention) concluded that studies exploring the effect of vitamin K supplementation (both PK [with doses ranging from 500 μg to 2 mg daily] and MK‐7 [with doses ranging from 90 to 2000 μg daily]), on vascular calcification, atherosclerosis and arterial stiffness remain inconsistent (Vlasschaert et al. 2020). This ambiguity may be attributed to the length of intervention, clinical heterogeneity between population groups (e.g., patients with CKD or diabetes), the dose and/or vitamin K isoform supplemented (PK vs. MK‐7), and the difference in vascular calcification outcomes measured (e.g., CAC and AVC). A subsequent RCT provided novel insights (*n =* 149 patients with diabetes mellitus, mean ± SD age: 65.5 ± 6.8 years, 66.4% male, with a coronary calcium score ≥ 10 Agatston units) where PK supplementation (10 mg/day for 3 months) inhibited the development of newly calcified lesions (identified via 18F‐NaF PET) within both the aortic (OR 0.27 95% CI 0.08–0.94) and coronary (OR 0.35 95% CI 0.16–0.78) arteries (Bellinge et al. 2022). These findings suggest that PK may be particularly useful at early, modifiable disease stages to potentially slow vascular calcification progression. However, as similarly reported within other reviews (Geng et al. 2023; Lees et al. 2019; Vlasschaert et al. 2020), inconsistency among the present literature remains and conclusions cannot yet be drawn.

While clinical trials typically provide supplemental vitamin K, the relationship between dietary vitamin K intakes and vascular calcification (often subclinical and asymptomatic) has been explored in observational studies. In the Rotterdam study cohort (*n =* 4807 61.8% female, aged ≥ 55 years), higher MK intakes (the highest [median 15.1 μg/day] compared to the lowest [median 40.9 μg/day] tertile) were associated with a lower risk of severe abdominal aortic calcification (AAC [OR 0.48 95% CI 0.32–0.71]), but not moderate AAC (Geleijnse et al. 2004). Similarly, in the Predictors of Response to Cardiac Resynchronization Therapy cohort (*n =* 564 Dutch women, mean ± SD age: 67 ± 5 years), a lower prevalence of CAC (PR 0.80 95% CI 0.65–0.98) was associated with higher energy‐adjusted MK intakes (quartile 4 [mean 48.5 μg/day], compared to quartile 1 [mean 18.0 μg/day]) (Beulens et al. 2009). No associations for PK intakes were reported in these studies (Beulens et al. 2009; Geleijnse et al. 2004). Conversely, in the Danish Diet, Cancer, and Health (DDCH) cohort (*n =* 55,545, 54% female, median [IQR] age: 56 [52–60] years), a lower risk of incident aortic valve stenosis was reported (HR 0.77 95% CI 0.63–0.94) when comparing the highest to the lowest PK intakes (quintile 5 [median 192 μg/day], vs. quintile 1 [median 57 μg/day]) (Schultz et al. 2024).

Various reviews have considered dietary vitamin K intakes with clinical CVD outcomes (Chen et al. 2019; Maresz 2015; Palmer et al. 2020; Shea et al. 2021). For example, a 2019 systematic review and meta‐analysis explored the link between vitamin K (both PK and MK) intakes, long‐term CVD events (including CVD mortality, stroke, nonfatal MI, fatal CHD, and total CHD) and all‐cause mortality, using data from seven cohort studies (Chen et al. 2019). The authors reported that higher dietary intake of both PK and MK were associated with a lower risk of CHD events (PK: pooled HR 0.92 95% CI 0.84–0.99 and; MK: pooled HR 0.70 95% CI 0.53–0.93), but not with other CVD or all‐cause mortality outcomes (Chen et al. 2019). Many reviews of observational studies have highlighted a cardioprotective benefit of higher MK intakes, but not PK intakes (Akbulut et al. 2020; Halder et al. 2019; Maresz 2015; Schwalfenberg 2017). Yet, as previously discussed, limitations to estimating population MK intakes remain, a barrier that ought to be considered when interpreting the findings of observational studies. More recently, results from a 2024 study within the Perth Longitudinal Study of Ageing Women (PLSAW) (*n =* 1436, mean ± SD age; 75.2 ± 2.7 years) reported higher PK intakes (quartile 4 [median 119.3 μg/day], compared to quartile 1 [median 49.1 μg/day]) were associated with a lower risk of both all‐cause (HR 0.66 95% CI 0.51–0.86) and CVD mortality (HR 0.61 95% CI 0.41–0.92) (Dupuy et al. 2024). Similarly, in the DDCH cohort (*n =* 56,048, 47.6% male, median [IQR] age; 56 [52–60] years), lower risks of both all‐cause (HR 0.76 95% CI 0.72–0.79) and CVD mortality (HR 0.72 95% CI 0.66–0.79) were observed with higher intakes of PK (Palmer, Bellinge, et al. 2021). Interestingly, both studies reported a plateau of benefit around PK intakes of ~80–100 μg/day (Dupuy et al. 2024; Palmer, Bellinge, et al. 2021). Although serum biomarkers such as MGP were not considered (Dupuy et al. 2024; Palmer, Bellinge, et al.^2021^ ), these findings indicate a threshold, beyond which higher PK intakes may not provide further benefit. Noteworthy, studies observing no benefits of PK for CVD‐related outcomes typically report vitamin K intakes of their reference group (and/or overall cohort) that are considerably higher than where this plateau occurred (Geleijnse et al. 2004; Haugsgjerd et al. 2020; Zwakenberg et al. 2017). A separate study in the DDCH cohort also reported that higher intakes of both PK (quintile 5 [median 192 μg/day], compared to quintile 1 [median 57 μg/day] and MKs quintile 5 [median 77 μg/day], compared to quintile 1 [median 23 μg/day]) were associated with lower risks of hospitalizations related to atherosclerotic cardiovascular diseases, including CHD (PK: HR, 0.86 95% CI 0.80–0.93, and MKs: HR 0.86 95% CI 0.80–0.92), stroke (PK: HR 0.83 95% CI 0.75–0.91, and MKs: HR 0.87 95% CI 0.79–0.95), and peripheral arterial disease (PK: HR, 0.66; 95% CI 0.58–0.75, and MKs: HR 0.88 95% CI 0.78–0.99) (Bellinge et al. 2021). However, MR studies have yielded conflicting results regarding circulating PK levels and CVD risk. For instance, one MR analysis indicated an increased risk of CHD with higher circulating PK (Schooling 2016), another found no causal link between circulating PK and CHD (Zwakenberg et al. 2020), and a third reported a higher risk of large artery atherosclerotic stroke with higher circulating PK (Larsson et al. 2018). These discrepancies may stem from the reliance on a single biomarker, such as circulating PK, primarily reflecting recent dietary intake, which may not adequately represent overall vitamin K status, as described above (Shea and Booth 2016).

The therapeutic potential of vitamin K in relation to cardiovascular health appears promising. However, ambiguity remains and much of the current evidence relies on observational studies, with conflicting findings from RCTs with relatively short‐intervention periods and limited MR studies. To strengthen and consolidate our understanding in this area, long‐term well‐powered clinical trials are warranted, especially in populations with habitually low vitamin K intakes.

## 3 Vitamin K and Type 2 Diabetes

The relationship between vitamin K and type 2 diabetes (T2D) has been reviewed in detail previously (Lacombe and Ferron 2024; Varsamis et al. 2021), with recent findings providing new insights into this connection. Here we provide a summary of recent findings and potential mechanisms through which vitamin K may influence T2D risk. Beyond its established role in bone health, vitamin K is thought to lower the risk of T2D (Beulens et al. 2010; Rasekhi et al. 2015; Zwakenberg et al. 2019), through pathways involving OC, a protein increasingly recognized for its metabolic regulatory functions (Levinger et al. 2017; Lin et al. 2020; Smith et al. 2024). While two studies demonstrated that dietary vitamin K supplementation improves insulin sensitivity, lowers ucOC, and increases carboxylated osteocalcin (cOC) (Choi et al. 2011; Yoshida et al. 2008), other studies have shown no effect (Tacey et al. 2021), or an increase in blood glucose and insulin resistance with reduced ucOC (Alfadda et al. 2013; Parker et al. 2019). This suggests that additional mechanisms may contribute to improved glucose homeostasis with higher vitamin K intake. A potential mechanism may be via its influence on pancreatic β‐cells (Lacombe et al. 2023), where the VKDP endoplasmic reticulum Gla protein (ERGP) present in such cells may regulate metabolic function by maintaining calcium flux as well as preventing physiological stress and dysfunction (Lacombe et al. 2023).

Vitamin K may also prevent insulin resistance (Juanola‐Falgarona et al. 2013; Rehman and Akash 2016), by inhibiting inflammation (Nakajima et al. 2011; Sikkens et al. 2013), particularly by reducing cytokine production (Ohsaki et al. 2010). This anti‐inflammatory property of vitamin K could contribute to improved glucose regulation, parallel to the potential benefits reported for cardiovascular health. In cellular animal studies, MK‐4 exhibits insulinotropic effects similar to incretins by suppressing glucagon production (Drucker 2006) and lowering blood glucose (Ho et al. 2019).

To date, four RCTs have investigated vitamin K supplementation in various populations, including patients with T2D (Karamzad et al. 2020; Rahimi Sakak et al. 2021), females who are prediabetic (Rasekhi et al. 2015), and healthy older adults (Yoshida et al. 2008). Two studies, providing MK‐7 capsules (200 μg/day and 180 μg two times a day, respectively) to patients with T2D (60 and 68 participants, respectively) found significantly lower fasting blood glucose and HbA1c compared to placebo after 3 months (Karamzad et al. 2020; Rahimi Sakak et al. 2021). Another trial conducted in females with prediabetes provided PK capsules (1000 μg/day for 4 weeks) and reported significant decreases in insulin sensitivity index, insulin level, and 2‐h post‐oral glucose tolerance level, though no effect on insulin resistance was observed (Rasekhi et al. 2015). Another trial among 355 healthy participants showed reduced HOMA‐IR and plasma insulin concentration among males after PK supplementation (500 μg/day for 3 years), with no significant difference in females (Yoshida et al. 2008). The authors speculated that adipose tissue, which is generally higher in women compared to men, might modulate the response to vitamin K supplementation, rendering vitamin K less available in women who were more obese. However, this hypothesis should be interpreted with caution as 500 μg/day still represents a very large dosage of PK. It is also uncertain whether these improvements in insulin regulation are sustained. As described above, dietary sources and bioavailability differ between PK and the MKs, which limits the comparability of findings from PK supplementation to those of MK supplementation. From a dietary perspective, a cross‐over design RCT involving individuals with untreated grade 1 hypertension examined the effect of vegetables rich in PK on bone metabolism (Sim et al. 2020) and blood glucose (Blekkenhorst et al. 2018). Following the 4‐week intervention of a high vitamin K diet, no significant difference in fasting plasma glucose was observed among high responders (suppression of ucOC ≥ median [≥ 30%]) (Blekkenhorst et al. 2018). Two separate intervention studies have also shown a reduction in ucOC levels following PK supplementation (1000 μg/day for 12 months and 500 μg/day for 21 days, respectively) (Centi 2015; Kumar et al. 2010). However, these reductions did not correspond with significant changes in fasting glucose, insulin concentration, and HOMA‐IR (Centi 2015; Kumar et al. 2010). Such findings suggest that the suppression of ucOC through vitamin K may not directly impact glycemic control, perhaps as vitamin K itself acts on the pancreas, as described above.

To the best of our knowledge, five cohort studies have examined the association between vitamin K intake and T2D incidence (Asadipooya et al. 2015; Beulens et al. 2010; Eshak et al. 2019; Ibarrola‐Jurado et al. 2012; Pokharel et al. 2023). A recent systematic review of these studies reported that the highest versus the lowest vitamin K intakes are associated with a lower risk of T2D (HR 0.79 95% CI 0.71–0.88) (Qu et al. 2023). However, this review did not differentiate between PK and MKs, likely due to limited data on MKs in food databases, and the scarcity of studies investigating the association between MKs and T2D (Beulens et al. 2010). Of the studies included in the review, only two differentiated between PK and MK intakes. Both studies observed a lower risk of T2D with higher intakes of PK ([HR 0.81 95% CI 0.66–0.99] (Beulens et al. 2010), and [HR 0.84 95% CI 0.71–0.99] [Ibarrola‐Jurado et al. 2012]), and a lower risk for higher MK intakes was also observed (HR 0.93 95% CI 0.87–1.00) (Beulens et al. 2010). Subsequently, in the DDCH cohort (*n =* 54,787, 52.7% female, median age; 56 years), a lower risk of diabetes (HR 0.69 95% CI 0.64–0.74) for participants with the highest PK intakes (quintile 5 [median 191 μg/day]), compared to those with the lowest intakes (quintile 1 [median 57 μg/day]), over 21 years of follow‐up was reported (Pokharel et al. 2023). A MR study utilizing genetic data from three large cohorts (*n =* 620,983) also suggested a protective association between genetically predicted circulating PK concentrations and the risk of T2D (Zwakenberg et al. 2019). This study used four SNPs known to predict circulating PK levels and found that higher genetically predicted PK concentrations were associated with a reduced risk of T2D (RR 0.93 95% CI 0.89–0.97, *I* ^2^ = 0.3%, *p =* 0.96). However, it is important to note, as discussed in the previous CVD section, that circulating PK reflects recent dietary intake (~24–72 h) and may not translate to habitual dietary vitamin K intake (Combs and McClung 2016; Dam et al. 2015; Schurgers and Vermeer 2000).

In the PREDIMED (Prevención con Dieta Mediterránea) study, participants with the greatest increase in intakes of PK over 1 year had significantly improved levels of biomarkers of diabetes and inflammation (Juanola‐Falgarona et al. 2013). The reductions were observed in glucose‐dependent insulinotropic polypeptide (12.9%), interleukin‐6 (27.9%), tumor necrosis factor (26.9%), visfatin (24.9%), leptin (10.3%), ghrelin (15%), and glucagon‐like peptide‐1 (17.6%). Similarly, a Dutch study reported high MK intakes and higher vitamin K status (indicated by low plasma dp‐ucMGP) were associated with a lower prevalence of metabolic syndrome, though no association was observed with PK intakes (Dam et al. 2015). The differences in associations in this study may be due to the varying absorption rates of vitamin K forms, with MK being more available for the carboxylation of MGP (Beulens et al. 2010). While most of the existing observational research underscores the beneficial role of PK in the diet for diabetes, limited attention has been given to the role of dietary MK, with only two studies exploring this (Beulens et al. 2010; Dam et al. 2015). Distinguishing between dietary sources rich in these vitamin K forms may be important for establishing dietary recommendations, yet complicated to disentangle, as high MK intakes may not align with a healthy diet due to being derived primarily from animal‐based foods.

In summary, the precise mechanism by which vitamin K influences glucose homeostasis is still unclear. It appears that the vitamin K cycle itself, and not just its dependent proteins, influences glucose regulation, potentially through effects on pancreatic β‐cells (Lacombe et al. 2023). This activation pathway may explain why some human studies report that the reduction in ucOC with vitamin K supplementation did not impair glucose control. Given the observed discrepancies in human studies with biomarkers like OC (Lin et al. 2018), the use of tissue‐specific biomarkers could provide targeted insights into the mechanistic relationship between vitamin K and T2D. Additionally, there may be other Gla proteins that help protect pancreatic β‐cell function (Lacombe and Ferron 2024). However, further human studies are crucial to support the protective effects of vitamin K on pancreatic β‐cell function observed in preclinical work. Future research should also consider the differences between PK and MK bioavailability and target the differential metabolic effects of vitamin K among population subgroups, including those with T2D and healthy at‐risk individuals.

## 4 Vitamin K and Pulmonary Diseases

Research on the role of vitamin K for pulmonary health is scarce. The hypothesis that vitamin K plays a role in pulmonary health stems from the relatively high expression of MGP, a VKDP known to be a potent inhibitor of soft‐tissue calcification (Schurgers et al. 2013), including in lung tissue (Fraser and Price 1988). The smaller size of MGP allows it to function within structures like elastin (Price et al. 2009), an essential structural protein in the lungs, impeding its calcification and subsequent degradation (Piscaer et al. 2023). Chronic obstructive pulmonary disease (COPD) is a disease characterized by accelerated elastic fiber breakdown in the lungs (Shifren and Mecham 2006). Patients with COPD have a reduced forced expiratory volume in 1 s (FEV_1_) and, although not to as great an extent, forced vital capacity (FVC) meaning a reduced FEV_1_/FVC ratio. Compared to healthy people, patients with COPD exhibit higher circulating levels of dp‐ucMGP (indicating lower vitamin K status) (Piscaer et al. 2019). The exact reasons for this remain uncertain, but it may be explained by reduced vitamin K intake, genetic factors affecting vitamin K recycling rates, or increased vitamin K utilization to counteract elastin degradation in patients with COPD (Piscaer et al. 2017). Higher levels of dp‐ucMGP have been shown to be associated with higher rates of elastic fiber degradation in patients with COPD and higher mortality risk, although whether degradation was occurring in the lungs and/or the vasculature could not be determined (Piscaer et al. 2019). Similar findings from two other cross‐sectional studies have been reported. Among 17,681 participants, adhering to the AI set by the National Institutes of Health for vitamin K (90 and 120 μg/day, for women and men, respectively) was associated with lower odds of emphysema (OR 0.61 95% CI 0.40–0.92) (Shen et al. 2020). While, among 4092 individuals, those with lower vitamin K status (reflected by higher levels of dp‐ucMGP) had poorer lung function (indicated by both a lower FEV_1_ and FVC) and a higher odds of having COPD (OR 2.24 95% CI 1.53–3.27), wheezing (OR 1.81 95% CI 1.44–2.28) and asthma (OR 1.44 95% CI 1.12–1.83) (Jespersen et al. 2023). In a case study, at least four out of nine family members had idiopathic pulmonary fibrosis, and genetic analyses pointed at variant alleles associated with low vitamin K status, including VKORC1 (Wijnen et al. 2019). Beyond its effects on VKDPs, such as MGP, vitamin K is also hypothesized to influence pulmonary health through VKDP‐independent pathways, including anti‐ferroptosis, anti‐inflammatory, and antioxidant mechanisms (Piscaer et al. 2023; Wang et al. 2023).

Given the role of coagulation in the pathophysiology of COVID‐19, the pandemic sparked an interest in vitamin K. It has been hypothesized that COVID‐19 may precipitate vitamin K deficiency as, theoretically, extrahepatic vitamin K may become depleted if it is utilized to a greater extent for carboxylation of pulmonary MGP and coagulation factors during infection (Janssen et al. 2021). This hypothesis is supported by observations that patients with severe COVID‐19 often develop comorbidities associated with reduced vitamin K status (Dofferhoff et al. 2021). For example, compared to controls, patients with COVID‐19 have higher levels of dp‐ucMGP (Dofferhoff et al. 2021; Linneberg et al. 2021), indicating insufficient extrahepatic vitamin K. Furthermore, in patients with COVID‐19, a lower vitamin K status was related to a higher rate of elastic fiber degradation (Dofferhoff et al. 2021), and mortality in age‐and sex‐adjusted analyses (Linneberg et al. 2021). However, an RCT examining the effect of 999 μd/day MK‐7 in COVID‐19 patients reported no effects on desmosine levels—a marker of elastic fiber degradation—despite improved vitamin K status (Visser et al. 2024). While these findings suggest a potential link between COVID‐19 and vitamin K deficiency, there are still numerous knowledge gaps that warrant further investigation.

While suggestive, these findings do not establish a causal link between vitamin K deficiency and respiratory health. Currently, no prospective studies have investigated the relationship between vitamin K intake or status and the development of respiratory diseases. In addition, no clinical trials have been performed to examine whether vitamin K supplementation can improve lung function, or whether there is a direct effect of vitamin K on lung health. Thus, while the association between vitamin K and lung health appears promising, further research is needed to elucidate its precise role and therapeutic potential in respiratory diseases.

## 5 Vitamin K and Musculoskeletal Health

Recent reviews on vitamin K and musculoskeletal health have focused on supplemental vitamin K (PK or MK) on BMD/osteoporosis and fractures (Fusaro et al. 2020; Tsugawa and Shiraki 2020), with less focus on muscle function (Alonso et al. 2023), a major risk factor for falls. Considering the key role diet plays in regulating vitamin K status, exploring the impact of dietary PK and/or MK intake on musculoskeletal outcomes is crucial, especially to support effective public health messaging.

Vitamin K insufficiency has been linked to poor muscle and bone health (van Ballegooijen et al. 2018). In the Longitudinal Aging Study Amsterdam (LASA, *n =* 633, aged 55–65 years, 54% female), compared to individuals with the lowest dp‐ucMGP (Tertile 1), those with the highest dp‐ucMGP (Tertile 3) had weaker hand grip strength (~1.1 kg) and smaller calf circumference (~0.5 cm) (van Ballegooijen et al. 2018). However, dp‐ucMGP was not related to the decline in these measures over 13 years. Such results may be related to the influence of vitamin K on neuromuscular as well as vascular function, which are critical to physical performance (van Ballegooijen et al. 2018). Consequently, vitamin K may play a role in preventing sarcopenia (low muscle mass and strength). This hypothesis is further supported by other work from the LASA cohort where higher dp‐ucMGP (Tertile 2 and 3 vs. 1) was associated with up to 75% greater odds of frailty over the next 13 years (Machado‐Fragua et al. 2020). This is important as frailty, when quantified using cumulative deficits across multiple health domains (psychosocial, physical, cognition, and comorbidities), is also reported to be strongly related to long‐term injurious falls, fractures, and mortality risk (including CVD mortality) (Dent et al. 2024).

The ratio of undercarboxylated OC to total OC (ucOC:tOC) is regarded as a marker of circulating vitamin K, with a higher ucOC:tOC ratio indicating lower vitamin K status (Sim et al. 2020). In 1261 older women (mean age: 75 years) from the PLSAW cohort, higher ucOC:tOC (> 0.47) was associated with greater risk for an injurious fall over 14.5 years (HR 1.31 95% CI 1.09–1.57) (Smith et al. 2021). A higher ucOC:tOC was also associated with a greater fear of falling and poorer physical function (timed‐up‐and‐go test), including its decline over 5 years (Smith et al. 2021). Similarly, higher ucOC:tOC (> 0.47) was associated with a higher risk of fracture‐related hospitalizations (Sim et al. 2022). While these findings highlight the importance of vitamin K status, reflected by ucOC:tOC for musculoskeletal health, for public health messaging it is also crucial to determine the approximate vitamin K intake required for such benefits to be observed. Indeed, a nadir in the relative hazards for both falls (~27%) (Sim et al. 2023), and fractures (as well as hip fractures [49%]) was observed at dietary intakes of ≥ 100 μg/day of PK (Sim et al. 2022). Notably, PK intake was inversely associated with ucOC:tOC in this cohort (*ρ* = −0.12, *p* < 0.001). Importantly, if ucOC:tOC has a key role in musculoskeletal health, this can be quite rapidly altered using diet. For example, a post hoc analysis from a randomized‐controlled trial of 30 middle‐aged adults found that ucOC:tOC was lowered by 19% in just 4 weeks by consuming 200 g (or 3–5 serves) per day of PK‐rich green leafy vegetables (Sim et al. 2020). This work also suggested that vitamin K recommendations < 100 μg/day may not support the maximal carboxylation of OC (Sim et al. 2020).

When considering dietary PK and fractures, comparable results have also been reported in the Hordaland Health Study (*n =* 2807, 71–75 years, 56% female) (Apalset et al. 2011), The Nurses' Health Study (*n =* 72,327 female, aged 38–63 years) (Feskanich et al. 1999), and the Framingham Heart Study (*n =* 888, mean age ~75 years, 62.2% female) (Booth et al. 2000). In all of the aforementioned studies, no associations with MK intakes were reported. This may be compounded by the current lack of comprehensive MK food databases to estimate dietary intake, as well as the source of PK often being plant‐based “healthier foods”, compared to meats and cheeses for the MKs (Palmer et al. 2020). To the best of our knowledge, only one study to date (*n =* 2944, age; ≥ 65 years, 55.5% male) found no relationship between dietary PK and fractures (Chan et al. 2012). However, the low fracture incidence (hip fractures ~1.6%) and/or very high median intake of PK (~240 μg/day), may explain the null associations (Chan et al. 2012). Collectively, such results are supported by a meta‐analysis of observational studies (*n =* 5 studies, 80,982 participants) finding that, compared to the lowest intakes, the highest PK intakes are associated with 22% lower fracture risk (RR 0.78 95% CI 0.56–0.99; *I* ^2^ = 59.2%, *p‐*heterogeneity = 0.04) (Hao et al. 2017). To the best of our knowledge, no MR studies have been undertaken to determine the effect of vitamin K status on falls and fractures, with only one study considering osteoarthritis. Here, utilizing four SNPs as genetic variants (*n =* 177,517 European individuals), no association between genetically predicted circulating PK and risk of osteoarthritis was reported (OR 0.98 95% CI 0.96–1.01) (Zhao et al. 2022). Clearly, huge scope exists for more research to be undertaken in this area.

Despite promising observational studies for dietary PK and musculoskeletal outcomes, clinical trials to date have typically opted to use supplemental MKs (e.g., MK‐4, MK‐7), and are summarized in detail previously (Alonso et al. 2023). This may be attributed to the higher bioavailability of MKs compared to PK, making it a more attractive option. Of note, a 2017 meta‐analysis of 19 clinical trials (*n =* 6759) supplementing with MKs reports no effect on BMD and vertebral fractures in women. However, in subgroup analyses that only included high‐risk populations, such as postmenopausal women and women who are osteoporotic, a beneficial effect for clinical fractures was observed (Huang et al. 2015). Specifically, in a sensitivity analysis where studies with high heterogeneity were excluded, a lower relative risk (RR 0.50 95% CI 0.33–0.74) for fracture incidence was reported with MK supplementation (Huang et al. 2015). Three separate meta‐analyses were subsequently undertaken in this area, with an additional two clinical trials exploring a combination of calcium, vitamin D, and bone‐active medications such as bisphosphonates (Kuang et al. 2020; Moore et al. 2023; Salma et al. 2022; Zhou et al. 2022). There appears to be some evidence for supplemental MKs to maintain/support BMD at different sites (e.g., femoral neck, vertebrae) and perhaps play a role in lowering fracture risk, especially in osteoporotic individuals. However, caution should be exercised as the literature is hampered by issues with the scientific integrity of a number of vitamin K supplementation trials conducted by a specific group (Kupferschmidt 2018). Of note, MKs continue to be approved in Japan for the treatment of osteoporosis since 1995 (Orimo et al. 2012). In contrast, the National Institute of Health currently highlights uncertainty regarding the benefits of vitamin K for fracture prevention (National Institutes of Health 2021). The International Osteoporosis Foundation has not only acknowledged the importance of vitamin K for bone mineralization, but also raises ambiguity when describing the link between low vitamin K levels, poor BMD, and fracture (International Osteoporosis Foundation 2024). Clearly, well‐powered clinical trials are required to determine if vitamin K truly plays an important role in musculoskeletal health.

## 6 Vitamin K and Cancer

Research on the antitumor properties of vitamin K has been underway since 1947, with the effectiveness of vitamin K against cancer believed to be due to the cytotoxic effect of quinone (Lamson and Plaza 2003). To date, nine epidemiological studies from six cohorts have examined associations between dietary vitamin K intake and cancer. Among 24,340 participants of the EPIC‐Heidelberg cohort (47% male, aged 35–64 years), the highest, compared to the lowest, intakes of MKs, but not PK, were associated with a lower risk of any cancer (HR 0.86 95% CI 0.73–1.01) and death with cancer as the underlying cause (HR 0.72 95% CI 0.53–0.98) (Nimptsch et al. 2010). Conversely, a higher intake of dietary PK was associated with a lower risk of cancer‐related mortality (HR 0.54 95% CI 0.30, 0.96) among 7216 participants from the PREDIMED study (Juanola‐Falgarona et al. 2014). Notably, participants who increased their intake of PK or MKs during follow‐up also had a lower risk of cancer (HR 0.64 95% CI 0.43–0.95, and HR 0.41 95% CI 0.26–0.64, respectively), when compared to individuals who decreased or did not change their intake (Juanola‐Falgarona et al. 2014). Neither PK nor MK intakes were associated with cancer‐related mortality in 33,289 participants of the EPIC‐Netherlands cohort (Zwakenberg et al. 2017). Conversely, among 56,048 participants of the Danish Diet Cancer and Health cohort, the highest intakes of PK were associated with a lower risk of cancer‐related mortality (HR 0.80 95% CI 0.75–0.86) in current and former, but not never smokers (Palmer, Bellinge, et al. 2021). Focusing on specific cancers, a higher intake of MKs, but not PK, was associated with a lower risk of prostate cancer (HR 0.65 95% CI 0.44–0.97) (Nimptsch et al. 2010), and advanced prostate cancer (HR 0.37 95% CI 0.16–0.88) in the EPIC‐Heidelberg cohort (Nimptsch et al. 2008). In a nested case–control study in this same cohort (250 prostate cancer cases and 494 matched controls), a higher ucOC:tOC ratio was associated with advanced‐stage and high‐grade prostate cancer (Nimptsch et al. 2009). Furthermore, there was evidence that the association between dietary MK intake and prostate cancer may be modified by the rs9934438 polymorphism of the VKORC1 gene, as evidence of an inverse association between higher dietary MK intakes and prostate cancer risk was seen in AA and AG but not GG carriers (Nimptsch et al. 2009). It is known that carriers of the A allele of this polymorphism have a higher expression of vitamin K epoxide reductase and therefore a more active vitamin K cycle (Wadelius et al. 2005). However, intakes of PK and MKs were not significantly associated with the risk of advanced, non‐advanced, and total prostate cancer (*n =* 2973 cases over a 8.5 year median follow‐up) among 48,090 male participants in the Prostate, Lung, Colorectal and Ovarian Cancer Screening Trial (Hoyt et al. 2019).

In accordance with findings from the EPIC‐Heidelberg cohort that higher MK intakes were associated with a lower risk of lung cancer (HR 0.38 95% CI 0.20–0.71) (Nimptsch et al. 2010), a higher intake of vitamin K was inversely associated with lung cancer (HR 0.67 95% CI 0.46–0.96) among 42,166 participants of the Japan Collaborative Cohort Study (Yan et al. 2023). It is worth noting that in the latter study, investigators could not distinguish between PK and MK, and nattō, a major source of MKs in the Japanese diet, was not included in the study's FFQ (Yan et al. 2023). Only one study has reported a positive association between vitamin K intakes and cancer; in 51,662 post‐menopausal women in the Prostate, Lung, Colorectal and Ovarian Cancer Screening Trial, total MK, but not PK, intake was associated with a higher risk of breast cancer (HR 1.26 95% CI 1.05–1.52) (Wang et al. 2021), However, as MKs are found abundantly in processed meats (e.g., salami, ham, and sausage) (Palmer, Koch, et al. 2021; Schurgers and Vermeer 2000), diet quality may contribute to this relationship.

In opposition to the mostly beneficial associations between dietary vitamin K intake and cancer incidence described above, there is evidence that vitamin K antagonists, such as warfarin, can lower cancer risk; in a meta‐analysis of nine observational studies (> 1.5 million patients), the use of VKAs for ≥ 6 months was associated with a lower risk of incident cancer (HR 0.84 95% CI 0.81–0.88) in comparison with control (< 6 months use or nonuse) (Shurrab et al. 2019). This reduction in risk is attributed to VKAs impact on cancer cell growth, angiogenesis, and host‐immune responses, mediated by vitamin K‐dependent proteins. However, an important limitation of observational studies on VKAs is confounding by indication. Specifically, VKAs are often prescribed to patients with underlying health conditions, such as atrial fibrillation or other CVDs, which may independently influence cancer risk. Additionally, confounding by treatment selection may occur, as patients prescribed VKAs may differ from those prescribed other anticoagulants, further complicating the interpretation of any association between VKA use and cancer.

Clinical trials on the efficacy of vitamin K in the prevention or treatment of cancer are scarce and all focus on hepatocellular carcinoma (HCC). In an RCT of 40 women diagnosed with viral liver cirrhosis, very high doses of MK‐4 (45 mg/day) for 2 years decreased the risk of developing HCC by 80% (RR 0.20 95% CI 0.04–0.91) (Habu et al. 2004). Since then, a further six studies have investigated the efficacy of postoperative therapy with MKs in patients with HCC (Hotta et al. 2007; Ishizuka et al. 2012; Kakizaki et al. 2007; Mizuta et al. 2006; Yoshida et al. 2011; Yoshiji et al. 2009). Although none of the studies reported any significant effect on overall survival after hepatic resection or local ablation, when the results from all seven studies were combined in a 2013 meta‐analysis, it was reported that MK therapy significantly reduced the rate of tumor recurrence at 2 and 3 years and significantly increased overall survival at 1, 2, and 3 years (Zhong et al. 2013).

Vitamin K has been shown to enhance the potency of various chemotherapeutic drugs against cancers of different origins in an additive or synergistic manner; this has been reviewed in detail previously (Gul et al. 2022). Mechanisms for vitamin K's anticancer and chemosensitizing effects include the inhibition of cancer cell proliferation, growth, differentiation, and survival through various mechanisms, including anti‐inflammatory and antioxidant effects, gene induction and regulation, and the inhibition of angiogenesis and cellular signaling pathways (Chen, Zhu, et al. 2023; Gul et al. 2022). The studies described above focus on natural forms of vitamin K; however, vitamin K3 and vitamin K5 may also act as chemosensitizers; this has been reviewed in detail previously (Gul et al. 2022). Whether any differences exist in the anticancer effects of the different forms of vitamin K remains unclear.

In summary, the intricate association between vitamin K and cancer presents a complex landscape in the literature, marked by inconsistent and even conflicting findings. While emerging evidence suggests vitamin K may have some role as a supplementary therapeutic agent in cancer management, further research is imperative to elucidate its full potential. Moreover, addressing the role of dietary vitamin K in primary cancer prevention necessitates future observational and genetic studies. These studies should incorporate refined methodologies to accurately estimate vitamin K intake and explore associations across diverse cancer types in varied populations with distinct dietary habits and sources of vitamin K.

## 7 Current Limitations and Directions for Future Work

The benefits of dietary vitamin K (both PK and MKs) for various health outcomes appears promising, yet ambiguity remains. Presently, it remains unclear whether a point of diminishing returns exists for the health benefits of vitamin K, particularly in individuals without apparent deficits in vitamin K status or dietary intake. Furthermore, RCTs routinely utilize supplemental vitamin K (PK or MK) and focus on short‐term surrogate outcomes, rather than long‐term disease incidence (e.g., BMD instead of fractures). As such, it is important for future studies to consider both supplemental and dietary vitamin K (both PK and MKs), and an broad array of outcomes, as part of study design. While providing valuable insights, current literature is largely observational in nature. Such studies typically adopt FFQs to estimate vitamin K intake that may result in misclassification bias, especially if not validated with appropriate biomarkers. Nevertheless, plasma PK levels have been shown to be positively correlated with PK intakes of up to ~200 μg/day (McKeown et al. 2002). Yet, the validity of an FFQ to estimate PK intakes beyond this level, as reported in many European cohorts (Chen et al. 2019; Geleijnse et al. 2004; Shea and Booth 2016; Zwakenberg et al. 2017), may be limited. Vitamin K content in foods is also known to vary by region (Palmer et al. 2020; Mladěnka et al. 2022), and few comprehensive food composition databases exist, particularly for the specific MKs (Shea and Booth 2016). Validating the efficacy of an FFQ to assess MK intakes is further complicated as reference biomarkers that are specific to each MK are yet to be established (Shea and Booth 2016). These limitations highlight the necessity for the development of comprehensive region‐specific vitamin K food databases and validated vitamin K status biomarkers (particularly for MKs). In addition, the implications of the food matrix, interindividual variability and the influence of genetics on vitamin K absorption and metabolism are not well understood. Current MR studies investigating the causal links between vitamin K and chronic diseases present divergent findings; some suggest a protective role of vitamin K in health (Liu et al. 2015; Wei et al. 2018; Zwakenberg et al. 2020, 2019), and others indicating potential harm (Larsson et al. 2018; Liu et al. 2015; Schooling 2016). On closer scrutiny, methodological flaws in these studies emerge, notably the reliance on genetic variants identified from small genome‐wide association studies using suboptimal measures of vitamin K status (Zhao et al. 2022; Zwakenberg et al. 2020). Furthermore, MR studies primarily derive their conclusions from genetic associations observed in European populations. Yet, ethnic or sex differences may influence vitamin K status, necessitating further research across a range of population groups. Genetic data could also be leveraged to identify individuals at higher risk of poor vitamin K status due to genetic predispositions, for whom increased dietary intake or supplementation may be particularly beneficial. Thus, the intricate interplay between genetic variants, vitamin K metabolism, and disease outcomes underscores the need for large, racially diverse GWAS to identify additional loci and refine our understanding of these relationships incorporating the influence of genetic polymorphisms. By leveraging genetic insights, we can develop more precise, genotype‐informed strategies to understand vitamin K metabolism, and improve health outcomes.

A well‐designed RCT is currently underway to improve our understanding of the benefits of vitamin K on health. The InterVitaminK trial, is a 3 year, double‐blind, proof‐of‐concept RCT designed to explore the effect of vitamin MK‐7 supplementation (333 μg/day vs. placebo) on vascular calcification progression (CAC, using Agatston score) in 450 participants with prevalent CAC, but without clinical manifestations of CVD (Kampmann et al. 2023). Secondary outcomes include a range of cardiovascular measures (e.g., total coronary plaque composition, arterial stiffness, AVC, and blood pressure), bone health (e.g., BMD, bone remodeling markers), pulmonary function (e.g., spirometry and lung tissue density), markers of diabetes (e.g., insulin resistance, lipid metabolism, and arthrometric measurement), and inflammatory biomarkers (Kampmann et al. 2023). Such trials are of paramount importance to address current knowledge gaps surrounding the potential roles of vitamin K in human health and to inform future research and clinical guidelines.

## 8 Conclusion

The potential benefit of vitamin K for a variety of health conditions and diseases appears promising. Yet, further work is needed due to remaining ambiguity in many conditions, including CVDs, diabetes, respiratory diseases, cancer, and musculoskeletal health. To understand the therapeutic potential and precise roles of vitamin K in human health, further research including large‐scale clinical trials, and broader MR and epidemiological studies are warranted. Addressing the current knowledge gaps is crucial to set dietary vitamin K requirements (for both PK and the MKs) to optimize health. To this end, based on current evidence, it is essential to continue promoting increased consumption of green leafy and cruciferous vegetables through public health messaging to ensure adequate PK intake. Specifically, consuming three or more serves a day of such vegetables will likely ensure adequate PK while also providing a range of other beneficial nutrients to support healthy aging.

## Author Contributions

**Montana Dupuy:** conceptualization (equal), writing – original draft (equal), writing – review and editing (equal). **Nicola P. Bondonno:** conceptualization (equal), writing – original draft (equal), writing – review and editing (equal). **Pratik Pokharel:** conceptualization (equal), writing – original draft (equal), writing – review and editing (equal). **Allan Linneberg:** writing – original draft (supporting), writing – review and editing (equal). **Itamar Levinger:** writing – original draft (supporting), writing – review and editing (equal). **Carl Schultz:** writing – original draft (supporting), writing – review and editing (equal). **Jonathan M. Hodgson:** writing – original draft (supporting), writing – review and editing (equal). **Marc Sim:** conceptualization (equal), writing – original draft (equal), writing – review and editing (equal).

## Conflicts of Interest

The authors declare no conflicts of interest.

## Data Availability

No primary research results have been included and no new data were generated or analyzed as part of this review.
